# Food and Water Insecurity and Functional Disability in Adults

**DOI:** 10.1001/jamanetworkopen.2025.1271

**Published:** 2025-03-20

**Authors:** Yankun Wang, Rodrigo X. Armijos, Sarah Commodore, Aurelian Bidulescu, M. Margaret Weigel

**Affiliations:** 1 Women and Children’s Hospital of Chongqing Medical University, Chongqing, China; 2 Chongqing Health Center for Women and Children, Chongqing, China; 3 Chongqing Research Center for Prevention and Control of Maternal and Child Diseases and Public Health, Chongqing, China; 4 Department of Environmental and Occupational Health, Indiana University-Bloomington School of Public Health, Bloomington; 5 Global Environmental Health Research Lab, Indiana University-Bloomington School of Public Health, Bloomington; 6 Department of Epidemiology and Biostatistics, Indiana University-Bloomington School of Public Health, Bloomington

## Abstract

**Importance:**

Evidence from diverse global populations suggests that household food insecurity (HFI) is associated with adult disability, but the association between household water insecurity (HWI) and disability remains understudied. Examinations of the joint association of dual HFI and HWI with disability are lacking.

**Objective:**

To explore independent and joint associations of HFI and HWI with functional disability in Ecuadorian adults.

**Design, Setting, and Participants:**

This cross-sectional study used data from the 2018 Ecuadorian National Health and Nutrition Survey, a nationally representative, population-based survey conducted in Ecuador. The study included adults (aged 18-99 years) with information on sociodemographic characteristics, household food and water security, and functional disability status. Data were collected in 2 waves of the survey, from November 2018 to January 2019 and June to July 2019. Statistical analysis was performed from May to December 2024.

**Exposures:**

HFI, WFI, or both.

**Main outcomes and Measures:**

The primary outcome was self-reported functional disability, assessed using the Washington Group Short Set on Functioning, a tool that uses a set of 6 questions to measure activity limitations.

**Results:**

Among 42 071 participants (mean [SE] age, 48.0 [0.1] years; 31 683 male [75.3%]; 1840 African descendant [4.4%], 5184 Indigenous [12.3%], and 35 047 Mestizo ethnic majority group [83.3%]), most lived in urban areas (26 164 participants [62.2%]). Exposure to HFI only was associated with an increased risk for any functional disability (adjusted relative risk [aRR], 1.44; 95% CI, 1.37-1.52), as well as sensory (aRR, 1.43; 95% CI, 1.35-1.52), physical (aRR, 1.56, 95% CI, 1.42-1.72), and cognitive (aRR, 1.78; 95% CI, 1.61-1.98) disabilities. HWI exposure only was associated with increased risk for any functional disability (aRR, 1.12; 95% CI, 1.06-1.20), as well as sensory (aRR, 1.17; 95% CI, 1.09-1.25), physical (aRR, 1.15; 95% CI, 1.05-1.26), and cognitive (aRR, 1.17; 95% CI, 1.03-1.34) disabilities. Exposure to dual HFI and HWI was associated with a greater increase in risk than HFI or HWI alone of any functional disability (aRR, 1.61; 95% CI, 1.50-1.72), as well as sensory (aRR, 1.65; 95% CI, 1.52-1.79), physical (aRR, 1.72; 95% CI, 1.59-1.87), and cognitive (aRR, 2.01; 95% CI, 1.76-2.29) disabilities.

**Conclusion and Relevance:**

In this study, dual exposure to HFI and HWI was associated with a greater increase in risk of any and specific functional disabilities compared with independent contributions of HFI and HWI alone. These findings highlight the importance of addressing HFI and HWI jointly rather than independently when conducting research on disability and other health outcomes and in designing policies and programs to protect at-risk adults and their households.

## Key Points

**Question:**

What are the independent and joint associations of household food insecurity (HFI) and household water insecurity (HWI) with functional disability in adults?

**Findings:**

In this cross-sectional study of survey data on 42 071 Ecuadorian adults, participants experiencing HFI or HWI had a significantly higher risk of any functional disability, including sensory, physical, and cognitive disabilities, compared with those with food and water security. Dual exposure to HFI and HWI was associated with greater increase in disability risk than each insecurity alone.

**Meaning:**

These findings suggest that addressing HFI and HWI together may be essential for reducing disability risk and supporting at-risk adult populations.

## Teaser

This cross-sectional study investigates independent and joint associations of household food insecurity and household water insecurity with functional disability among adults in Ecuador.

## Introduction

Food and water security are critical to human health, well-being, and survival. The United Nations (UN) recognizes both as basic human rights^1^ and essential to achieving the 2030 Sustainable Development Goals.^2^ Household food insecurity (HFI), defined as limited or uncertain availability of nutritionally adequate and safe foods or limited or uncertain ability to acquire acceptable foods in socially acceptable ways by households for their members,^3^ affects an estimated 2.4 billion people, or 29.6% of the global population.^4^ Household water insecurity (HWI), the inability to access and benefit from affordable, adequate, reliable, and safe water needed to support a healthy life,^5^ impacts an estimated 2 billion people, or 26% of the world population.^1^ Approximately 80% of people affected by HFI, HWI, or both live in low- and middle-income countries (LMICs).^1,4^

Risk factors associated with HFI and HWI include age and poverty–related sociodemographic and environmental inequities, such as low income, low education level, unemployment, ethnoracial minority status, having a household member with a disability, and living in a rural area or underresourced neighborhoods.^6,7,8,9,10^ HFI^8,11,12,13,14,15^ and HWI are associated with suboptimal diets, undernutrition, overnutrition, and poorer mental and physical health outcomes.^16,17,18,19,20^

Published evidence has associated HFI and HWI with disability. The World Health Organization (WHO) estimates that 1.3 billion people, or 1 in 6 people globally, live with a disability, with 80% residing in LMICs.^21^ Research shows that HFI is associated with increased risk of disability among adults in high-income^6,22^ and LMIC settings, such as Mexico,^23,24^ India,^15,25,26^ and sub-Saharan Africa.^27,28,29,30^ While most prior studies have focused on the association of existing disabilities with HWI,^31,32,33,34^ it is likely that HWI also contributes to the risk of sensory, physical, and cognitive disabilities in adults through similar pathways to those hypothesized for HFI.

The association of HFI with functional disability risk in adults is well documented across a diverse population, with emerging evidence associating HWI with similar risks. Studies suggest that HFI and HWI often co-occur^35,36^ and may interact in associations with adult nutrition and health outcomes.^20,36,37,38^ However, the potential joint association of HFI and HWI with functional disability in adults has not yet been explored, to our knowledge, presenting a critical knowledge gap with important public health implications. Furthermore, individual or dual associations of HFI and HWI with adult disability are understudied in Latin American population settings, a global region with a high prevalence of HFI,^4^ HWI,^39^ and disability.^40^

Thus, to explore the potential joint association of HFI and HWI with functional disability, we conducted a secondary analysis of data from a large, nationally representative survey to examine independent and joint associations of HFI and HWI with functional disability in Ecuadorian adults. Ecuador is a middle-income country characterized by a high national prevalence of food insecurity (37.3%)^4^ and inadequate access to safely managed water (33%),^2^ as well as high prevalence of functional disability (25%).^21^ Our working hypothesis was that exposure to HFI and HWI in adults would each be independently associated with the risk for functional disability and that their combined exposure would be associated with a greater increase in risk than their individual exposures.

## Methods

### Data and Study Population

The cross-sectional study analyzed data from the 2018 Ecuadorian National Health and Nutrition Survey (Spanish acronym, ENSANUT),^41^ a nationally representative, population-based survey conducted by the Ecuadorian National Institute of Statistics and Census. The survey used a probabilistic 2-stage sampling strategy to obtain data from a nationally representative sample of 43 311 households containing noninstitutionalized individuals aged 0 to 99 years; the sample had no missing data on dependent or independent variables or covariates.

Data collection took place in 2 waves of the survey, from November 2018 to January 2019 and June to July 2019. We used the *Personas* (Persons) and *Hogar* (Household) modules containing information on participant and household sociodemographic characteristics, HFI and HWI indicators, and functional disability in adults aged 18 to 99 years. After exclusion of participants younger than 18 years, the final analytical sample included 42 071 adults. Given that publicly available data used in the analyses were fully deidentified, the institutional review board of Indiana University Bloomington classified the study as “research not subject to human subject regulations.” This study followed the Strengthening the Reporting of Observational Studies in Epidemiology (STROBE) reporting guideline.

### Outcome Variables

The primary outcome of interest was self-reported functional disability in adults, assessed using the Washington Group Short Set on Functioning (WG-SS), a widely-used tool based on the WHO International Classification of Functioning, Disability and Health and focusing on activity limitations rather than explicitly referencing *disability*.^42^ The WG-SS includes 6 questions addressing sensory, physical, and cognitive disabilities. Specific WG-SS questions to assess sensory disabilities were difficulty in seeing even with glasses or difficulty in hearing even with hearing aids. Physical disabilities were difficulty in walking or going upstairs or difficulty in self-care, such as bathing or dressing. Cognitive disabilities were difficulty in remembering or concentrating and difficulty in communicating, such as understanding or being understood. Participants who reported any difficulty on these indicators were classified as having a limitation in that domain. We categorized participants into 3 disability domains (sensory, physical, and cognitive) based on reported difficulties. Individuals reporting any limitation were classified as having functional disabilities.

### Exposure Variables

HFI was assessed using the 8-question Food Insecurity Experience Scale developed by the UN Food and Agricultural Organization,^4^ which gathered data on food access difficulties during the previous 12 months. Responses were coded as *1* for affirmative and *0* for negative, with a total score ranging from 0 to 8. Participants with Food Insecurity Experience Scale scores of 1 or greater were classified as food insecure, while those with scores of 0 were classified as food secure.^43^

Household drinking water security was assessed using Joint Monitoring Programme indicators^44^ based on 4 key water indicators: water source, water accessibility, water availability, and perceived drinking water quality (safety) in the 2 weeks prior to the survey. Participants were classified as water secure if they responded positively to all 4 indicators and as water insecure if they responded negatively to 1 or more indicators.

We created a 4-category composite indicator for food and water security status. Categories were defined as follows: (1) food and water secure: households with no HFI or HWI, (2) HFI only: households with HFI but not HWI, (3) HWI only: households with HWI but not HFI, and (4) dual HFI and HWI: households with both HFI and HWI.

### Covariates

Data were collected on sociodemographic characteristics in the survey. These included participant age (years), sex (male or female), ethnicity (Indigenous, African descendant, and Mestizo) self-reported in the original survey, marital status (legally married, common-law union, single, divorced or separated, or widowed), formal education level (none, primary or basic education, middle school, high school, postsecondary technical school, or postsecondary undergraduate or graduate school), household asset score, residence site (urban or rural), and region of residence (Andean Highlands, Pacific Coast Lowlands, Ecuadorian Amazon, or Galapagos Islands). Ethnicity was assessed in this study to explore potential differences or disparities among ethnic groups.

### Statistical Analysis

Study data were analyzed using R statistical software version 4.3.2 (R Project for Statistical Computing) and Stata statistical software version 18.0 (StataCorp). All analyses were weighted using survey-provided sampling weights. Participant characteristics and other descriptive statistics are presented as number with percentage or mean with standard error (SE), as appropriate. We also conducted generalized linear model univariate analysis or the χ^2^ test, as appropriate, to initially examine the association of participant sociodemographic characteristics with the 4-category HFI and HWI composite indicator. We next constructed modified Poisson regression models with cluster standard error estimates for each functional disability category to analyze independent and joint associations of HFI and HWI with disability. Furthermore, we performed a sensitivity analysis by adding the interaction term for sex, wealth, and urbanicity with our HFI and HWI composite indicator. We also did a subgroup analysis stratified by sex, wealth, and urbanicity to explore potential differences across these factors. Adjusted models controlled for covariates, including participant age, sex, education level, marital status, ethnoracial group, urbanicity, region of residence, and household assets score. Findings from these analyses are reported as unadjusted relative risk (uRR) and adjusted relative risk (aRR) with their respective 95% CIs. A 2-tailed *P* < .05 was regarded as statistically significant. Data were analyzed from May to December 2024.

## Results

Among 42 071 participants (mean [SE] age, 48.0 [0.1] years; 31 683 male [75.3%]; 1840 African descendant [4.4%], 5184 Indigenous [12.3%], and 35 047 Mestizo ethnic majority group [83.3%]), two-thirds were legally married or in a common-law union (29 690 individuals [70.6%]) and most (26 164 individuals [62.2%]) lived in urban areas (Table 1). Most participants lived in the Andean Highlands (16 559 [39.4%]) or Pacific Coast Lowlands (14 615 individuals [34.7%]). Additionally, there were 6091 participants (14.5%) from HFI-only households, 15 146 participants (36.0%) from HWI-only households, and11 210 participants (26.6%) from households that were dually HFI and HWI. Food and water security status differed significantly by participant characteristic, including age, sex, household assets score, residence site, region of residence, education level, and marital status.

**Table 1.** Participant Sociodemographic Characteristics

Characteristic	Participants, No. (%)^a^					*P* value^b^
	Total (N = 42 071)	Food and water secure (n = 9624)	HFI only (n = 6091)	HWI only (n = 15 146)	Dual HFI and HWI (n = 11 210)	
Age, mean (SE), y	48.0 (0.1)	48.9 (0.3)	47.3 (0.4)	48.6 (0.2)	47.4 (0.3)	
18-39	16 972 (40.3)	3651 (37.9)	2576 (42.3)	5977 (39.5)	4768 (42.5)	<.001
40-59	15 987 (38.0)	3664 (38.1)	2227 (36.6)	5928 (39.1)	4168 (37.2)	
60-74	6551 (15.6)	1641 (17.1)	881 (14.4)	2366 (15.6)	1663 (14.8)	
≥75	2561 (6.1)	668 (6.9)	407 (6.7)	875 (5.8)	611 (5.5)	
Sex						
Male	31 683 (75.3)	7294 (75.8)	4316 (70.9)	11745 (77.5)	8328 (74.3)	<.001
Female	10 388 (24.7)	2330 (24.2)	1775 (29.1)	3401 (22.5)	2882 (25.7)	
Ethnoracial group						
African descendant	1840 (4.4)	345 (3.5)	300 (4.9)	583 (3.8)	612 (5.5)	<.001
Indigenous	5184 (12.3)	785 (8.2)	812 (13.3)	1432 (9.5)	2155 (19.2)	
Mestizo	35 047 (83.3)	8494 (88.3)	4979 (81.8)	13131 (86.7)	8443 (75.3)	
Household assets score, mean (SE)	19.1 (0.1)	21.1 (0.2)	16.9 (0.2)	21.7 (0.2)	15.4 (0.2)	<.001
Residence site						
Urban	26 164 (62.2)	6906 (71.8)	3874 (63.6)	9823 (64.9)	5561 (49.6)	<.001
Rural	15 907 (37.8)	2718 (28.2)	2217 (36.4)	5323 (35.1)	5649 (50.4)	
Region of residence						
Andean Highlands	16 559 (39.4)	5129 (53.3)	3040 (49.9)	4651 (30.7)	3739 (33.3)	<.001
Pacific Coastal Lowlands	14 615 (34.7)	2582 (26.8)	1810 (29.7)	5874 (38.8)	4349 (38.8)	
Ecuadorian Amazon	9110 (21.7)	1743 (18.1)	1218 (20.0)	3249 (21.4)	2900 (25.9)	
Galapagos Islands	1787 (4.2)	170 (1.8)	23 (0.4)	1372 (9.1)	222 (2.0)	
Formal education						
None	2013 (4.8)	354 (3.7)	363 (6.0)	507 (3.3)	789 (7.0)	<.001
Primary school or basic	17 608 (41.8)	3596 (37.4)	3107 (51.0)	5212 (34.4)	5693 (50.8)	
Middle school	1801 (4.3)	426 (4.4)	285 (4.7)	613 (4.1)	477 (4.3)	
High school	13 454 (32.0)	3099 (32.2)	1816 (29.8)	5271 (34.8)	3268 (29.1)	
Postsecondary technical school	936 (2.2)	258 (2.7)	97 (1.6)	429 (2.8)	152 (1.4)	
Postsecondary undergraduate or graduate education	6259 (14.9)	1891 (19.6)	423 (6.9)	3114 (20.6)	831 (7.4)	
Marital status						
Legally married	17 030 (40.5)	4257 (44.2)	2218 (36.4)	6419 (42.4)	4136 (36.9)	<.001
Common-law union	12 660 (30.1)	2502 (26.0)	1968 (32.3)	4392 (29.0)	3798 (33.9)	
Single	4017 (9.5)	931 (9.7)	597 (9.8)	1451 (9.6)	1038 (9.2)	
Separated or divorced	5453 (13.0)	1221 (12.7)	840 (13.8)	1927 (12.7)	1465 (13.1)	
Widowed	2911 (6.9)	713 (7.4)	468 (7.7)	957 (6.3)	773 (6.9)	

Abbreviations: HFI, household food insecurity; HWI, household water insecurity; SE, standard error.

^a^ Analyses are weighted using 2018 Ecuadorian National Health and Nutrition Survey–provided sampling weights.

^b^ *P* values were calculated for any difference across food and water security status, using analysis of variance (for continuous variables) or the χ^2^ test (for categorical variables).

**Figure 1.**
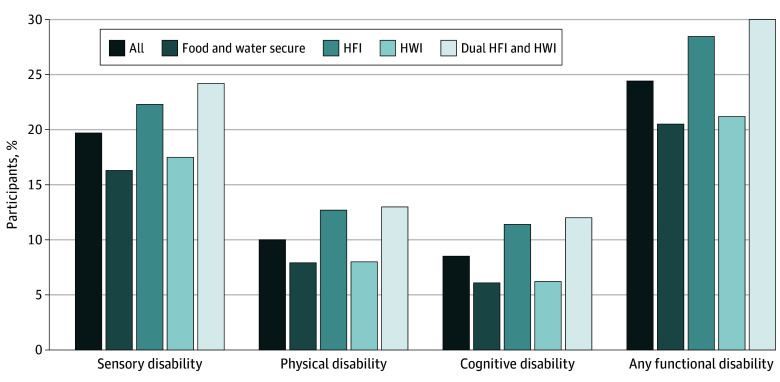
Prevalence of Functional Disability The prevalence of sensory, physical, cognitive, and any functional disability is shown by food and water security status. Percentages are shown of individuals with disabilities across 5 categories: overall population (all participants), individuals who were food and water secure, and those with household food insecurity (HFI) only, household water insecurity (HWI) only, and dual HFI and HWI. The prevalence of disabilities increased with HFI and HWI, with the highest rates observed in the dual HFI and HWI group across all disability types. Any functional disability showed the highest overall prevalence, particularly under dual HFI and HWI conditions.

Figure 1 compares the prevalence of participants who reported experiencing any sensory, physical, or cognitive disability and any functional disability further stratified by household food and water security status. The prevalence of the 3 specific sensory, physical, and cognitive functional disabilities or any functional disability was higher among all participants and those exposed to HFI only, HWI only, or dual HFI and HWI compared with their food- and water-secure counterparts.

**Figure 2.**
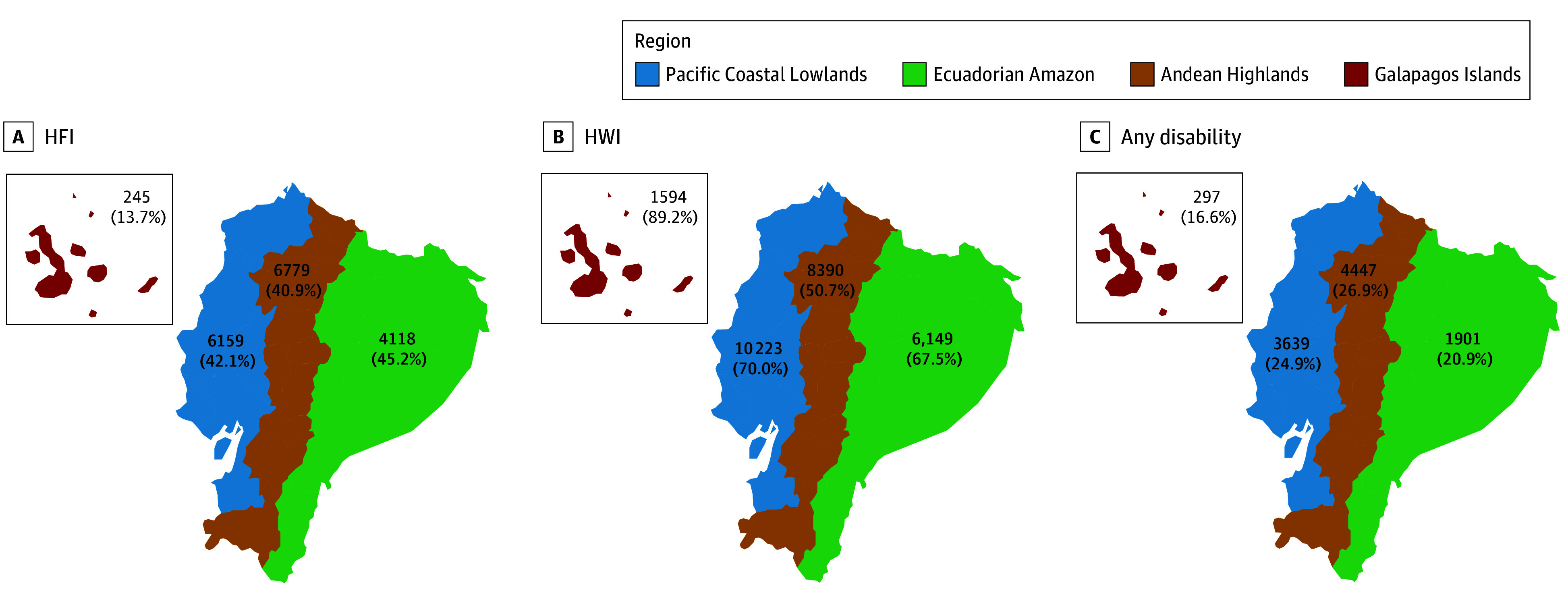
Distributions of Household Food Insecurity (HFI), Water Insecurity (HWI), and Disability Variations of HFI, HWI, and any functional disability across geographic regions are shown.

Figure 2 shows the spatial distribution of exposures and outcomes, highlighting variations of HFI, HWI, and any functional disability by geographic region. The prevalence of HFI was much lower in the Galapagos Islands, while HWI was higher. The prevalence of any functional disability was highest in the Andean Highlands (4447 participants [26.9%]), followed by the Pacific Coastal Lowlands (3639 participants [24.9%]), Ecuadorian Amazon (1901 of 9110 participants [20.9%]), and Galapagos Islands (297 of 1787 participants [16.6%]).

Table 2 displays findings from modified Poisson regression analyses examining unadjusted and adjusted associations of the 4-category composite HFI and HWI indicator with functional limitations. Unadjusted results showed that HFI-only exposure was associated with an increased risk of sensory (uRR, 1.36; 95% CI, 1.28-1.44), physical (uRR, 1.60; 95% CI, 1.45-1.77), and cognitive (uRR, 1.87; 95% CI, 1.66-2.11) limitations compared with being food and water secure. HWI-only exposure was also associated with an increased risk of sensory (uRR, 1.07; 95% CI, 0.98-1.18), physical (uRR, 1.00; 95% CI, 0.86-1.17), and cognitive (uRR, 1.01; 95% CI, 0.85-1.21) limitations. Furthermore, the magnitude of the associated increase in risk for participants dually exposed to HFI and HWI was higher for sensory (uRR, 1.48; 95% CI, 1.36-1.62), physical (uRR, 1.63; 95% CI, 1.45-1.84), and cognitive (uRR, 1.97; 95% CI, 1.71-2.27) limitations, as well as any functional limitation (uRR, 1.47; 95% CI, 1.36-1.58) than for HFI or HWI alone.

**Table 2.** Association of HFI and HWI With Functional Disability

Security status	Functional disability, RR (95% CI) (N = 42 071)^a^^,^^b^							
	Unadjusted				Adjusted^c^			
	Any sensory	Any physical	Any cognitive	Any functional^d^	Any sensory	Any physical	Any cognitive	Any functional^d^
Food and water secure	1.00 [Reference]	1.00 [Reference]	1.00 [Reference]	1.00 [Reference]	1.00 [Reference]	1.00 [Reference]	1.00 [Reference]	1.00 [Reference]
HFI only	1.36 (1.28-1.44)	1.60 (1.45-1.77)	1.87 (1.66-2.11)	1.39 (1.32-1.46)	1.43 (1.35-1.52)	1.56 (1.42-1.72)	1.78 (1.61-1.98)	1.44 (1.37-1.52)
HWI only	1.07 (0.98-1.18)	1.00 (0.86-1.17)	1.01 (0.85-1.21)	1.03 (0.94-1.13)	1.17 (1.09-1.25)	1.15 (1.05-1.26)	1.17 (1.03-1.34)	1.12 (1.06-1.20)
HFI and HWI	1.48 (1.36-1.62)	1.63 (1.45-1.84)	1.97 (1.71-2.27)	1.47 (1.36-1.58)	1.65 (1.52-1.79)	1.72 (1.59-1.87)	2.01 (1.76-2.29)	1.61 (1.50-1.72)

Abbreviations: HFI, household food insecurity; HWI, household water insecurity; RR, relative risk.

^a^ Analyses are weighted using 2018 Ecuadorian National Health and Nutrition Survey–provided sampling weights.

^b^ Data were analyzed using modified Poisson regression analysis.

^c^ Adjusted for participant age, sex, education level, marital status, ethnicity, urbanicity, region of residence, household asset score.

^d^ Any self-reported sensory, physical, or cognitive disability.

The adjusted model in Table 2 confirmed similar findings. Participants exposed to HFI only showed an increased risk of sensory (aRR, 1.43; 95% CI, 1.35-1.52), physical (aRR, 1.56; 95% CI, 1.42-1.72), cognitive (aRR, 1.78; 95% CI, 1.61-1.98), and any functional (aRR, 1.44; 95% CI, 1.37-1.52) limitations compared with the reference group. Participants exposed to HWI only had an increased risk of sensory (aRR, 1.17; 95% CI, 1.09-1.25), physical (aRR, 1.15; 95% CI, 1.05-1.26), cognitive (aRR, 1.17; 95% CI, 1.03-1.34), and any functional (aRR, 1.12; 95% CI, 1.06-1.20) limitations compared with the reference group. Furthermore, participants with dual HFI and HWI exposure had an increased risk of sensory (aRR, 1.65; 95% CI, 1.52-1.79), physical (aRR, 1.72; 95% CI, 1.59-1.87), cognitive (aRR, 2.01; 95% CI, 1.76-2.29), and any functional (aRR, 1.61; 95% CI, 1.50-1.72) limitations compared with the food and water secure reference group, and these increases in risk were greater than those associated with HFI or HWI alone.

Associations of HFI and HWI with functional disabilities stratified by sex, wealth, and urbanicity are presented in the eTable in Supplement 1. No significant differences were identified by interaction analysis when sex was added to food and water insecurity status. However, the interaction analysis revealed that household wealth interacted with the associations between HFI and HWI status and physical and cognitive disabilities. In addition, subgroup analysis showed that exposure to HWI only was associated with cognitive disability in urban areas (aRR, 1.20; 95% CI, 1.06-1.37) but not rural areas (aRR, 1.12; 95% CI, 0.96-1.30).

## Discussion

This cross-sectional study examined independent and joint associations of HFI and HWI with functional disabilities in adults using data from the large, nationally representative 2018 ENSANUT survey. To the best of our knowledge, it is one of few studies to report on independent associations of exposure to HFI and HWI with disability in Latin American adults. It is the first to examine the joint association of these 2 exposures with functional disabilities in adults, to our knowledge. We found that exposure to HFI only or HWI only was independently associated with an increased risk of any functional disability, as well as specific sensory, physical, and cognitive disabilities. The increase in risk was highest for HFI. Most importantly, dual exposure to HFI and HWI was associated with a greater increase in risk of functional disabilities beyond the independent contributions of HFI and HWI.

As we hypothesized, HFI only was independently associated with increased risk for overall functional disabilities and the 3 disability types examined in this study. Our findings align with those of studies across diverse populations, including findings for sensory (vision and hearing),^22,45,46,47,48^ physical (mobility),^22,26,49,50,51,52,53^ and cognitive (memory and concentration)^23,24,30,54,55,56,57,58^ difficulties.

As we had hypothesized, HWI only was also independently associated with increased risk for functional disabilities. The HWI-disability association is an emerging, yet understudied area. Unlike our study, which examined HWI as a risk factor associated with disability, previous research has focused on preexisting disability as a risk factor associated with HWI. These studies found that adults with disabilities often struggled to access adequate potable water for household use.^31^ A study in Vietnam, for example,^32^ found that more adults with HWI reported long-term or permanent difficulties in using their legs or bodies. It is suggested that adults with disabilities may face higher HWI risk owing to lower incomes, reduced labor market participation, and higher out-of-pocket expenses for health care, transportation, and other needs.^6,31,32,33,34,59,60^

Some pathways hypothesized for HFI may also explain how HWI may be associated with functional disability risk. These include lower dietary quality reported for food-insecure households.^18,61^ Other potential pathways include the ill effects of chronic water insecurity on hydration status, blood pressure, and kidney function.^20,62^ Furthermore, HWI, like HFI, can be a stressful experience, leading to an increased production of cortisol and other stress hormones via activation of the hypothalamic-pituitary-adrenal axis.^62^

In our study, more than one-quarter of participants lived in households with dual HFI and HWI, consistent with 2 prior studies.^63,64^ Interestingly, participants exposed to HFI and HWI had a much higher increase in risk of cognitive disability compared with other types of disabilities. This could be due to the combined stressors of HFI and HWI, which may disproportionately contribute to cognitive function through heightened psychological distress.^58^ Additionally, malnutrition and dehydration caused by HFI and HWI could impair basic and higher cognitive functions.^65^ This finding suggests that the dual deprivation may be associated with more severe cognitive disability than sensory or physical disabilities.

As we hypothesized, dual exposure to HFI and HWI was associated with a greater increase in risk for functional disabilities than either exposure alone. Due to a dearth of prior studies, we were unable to compare our finding on the association of dual HFI and HWI exposure with adult functional disability against previous findings. However, the general pattern aligns with recent findings reporting synergistic associations of dual HFI and HWI with poorer physical and mental health outcomes in adults living in Vietman^66^ and Kenya,^67^ mixed child and adult households in the Galapagos Islands,^64^ and Ecuadorian children.^63^ The apparent synergism between HFI and HWI in the association with adult disability likely operates through nutritional, sanitation and hygiene, and psychosocial pathways. Emerging evidence also suggests that HWI contributes to HFI in some environments.^37^ For example, low water supplies can reduce crop or food productivity in rural households, and limited drinking water can restrict the types of foods consumed.^20^

Although the Ecuadorian constitution explicitly guarantees the right to food and water security,^68^ the persistence of high rates of poverty, extreme poverty, and poverty-linked and environmental inequities continues to make many households at risk of HFI and HWI. Strengthening the enforcement of existing laws and policies, expanding conditional cash transfer and other antipoverty social programs, and investing in water and sanitation infrastructure could improve the food and water security situation for at-risk adults and their households.

### Limitations

This study has several potential limitations. The self-reported data on HFI, HWI, and functional disabilities may be subject to recall and social desirability biases. The cross-sectional design prevents establishing causality or temporality. While HFI, HWI, and dual HFI and HWI may be associated with increased risk for functional disabilities among adults, the associations could be bidirectional given that functional disabilities may also be associated with the risk of developing HFI^6,59^ or HWI.^34^ Additionally, reference periods for exposure and outcome indicators varied given that HFI was assessed over the past 12 months, HWI indicators had different time frames (eg, 2 weeks for drinking water accessibility and none for water source, access, and perceived safety), and functional limitations had no reference period, which may impact measurement validity and comparability. The generalizability of this study is limited to adults from Ecuador or similar Andean region countries given that we analyzed a single population. Furthermore, while prior research suggests that HFI and HWI may vary within communities,^37^ we were unable to assess community-level variation owing to a lack of community data. Additionally, while we adjusted for several known confounders, there could be unmeasured factors, like environmental contaminants (eg, lead, mercury, and pesticides) present in food and water, contributing to confounding.

## Conclusions

In this cross-sectional study examining the association of HFI and HWI exposure with functional disabilities in adults, we found that dual exposure to HFI and HWI was associated with a greater increase in risk for functional disabilities among adults beyond the independent contributions of HFI or HWI alone. Our findings highlight the importance of addressing food and water insecurity jointly rather than individually in research on disability and other health outcomes, as well as in the design and implementation of policies and programs aimed at protecting at-risk adults and their households.

## Notes

Supplement 1. **eTable.** Association of household food and water security status with functional disabilities stratified by sex, wealth, and urbanicity

Supplement 2.
Data Sharing Statement
