# Supplementary material for: Food and Water Insecurity and Functional Disability in Adults
Source: JAMA Netw Open. 2025 Mar 20;8(3):e251271. doi: 10.1001/jamanetworkopen.2025.1271 (PMC11926644; doi:10.1001/jamanetworkopen.2025.1271)
Supplement: Supplement 1. — eTable. Association of household food and water security status with functional disabilities stratified by sex, wealth, and urbanicity [file jamanetwopen-e251271-s001.pdf]

Supplemental Online Content

Wang Y, Armijos RX, Commodore S, Bidulescu A, Weigel MM. Food and water insecurity and functional disability in adults. *JAMA Netw Open*. 2025;8(3):e251271. doi:10.1001/jamanetworkopen.2025.1271

eTable. Association of household food and water security status with functional disabilities stratified by sex, wealth, and urbanicity

This supplemental material has been provided by the authors to give readers additional information about their work.

© 2025 Wang Y et al. *JAMA Network Open*.

eTable. Association of household food and water security status with functional disabilities stratified by sex, wealth, and urbanicity[a,b]

		Adjusted RR (95% CI)				P for interaction
		**Food & water secure**	**Food insecurity only**	**Water insecure only**	**Both food & water insecure**	
**Any sensory disabilities**						
Sex						0.398
	Male	1.00	1.41	1.13	1.63	
		Reference	(1.31, 1.52)***	(1.06, 1.21)***	(1.52, 1.74)***	
	Female	1.00	1.47	1.27	1.68	
		Reference	(1.33, 1.63)***	(1.16, 1.39)***	(1.53, 1.85)***	
Wealth						0.424
	Low	1.00	1.43	1.21	1.62	
		Reference	(1.29, 1.59)***	(1.09, 1.34)***	(1.47, 1.79)***	
	Middle	1.00	1.39	1.17	1.70	
		Reference	(1.25, 1.55)***	(1.06, 1.28)**	(1.55, 1.86)***	
	High	1.00	1.49	1.14	1.65	
		Reference	(1.33, 1.66)***	(1.05, 1.24)**	(1.50, 1.81)***	
Urbanicity						0.370
	Rural	1.00	1.39	1.14	1.57	
		Reference	(1.26, 1.55)***	(1.04, 1.25)**	(1.43, 1.71)***	
	Urban	1.00	1.44	1.17	1.69	
		Reference	(1.34, 1.56)***	(1.09, 1.25)***	(1.58, 1.81)***	
**Any physical disabilities**						
Sex						0.268
	Male	1.00	1.54	1.12	1.76	
		Reference	(1.37, 1.73)***	(1.01, 1.24)*	(1.59, 1.95)***	
	Female	1.00	1.59	1.19	1.65	

© 2025 Wang Y et al. *JAMA Network Open*.

		Reference	(1.40, 1.81)***	(1.06, 1.35)**	(1.46, 1.87)***	
Wealth						<0.001
	Low	1.00	1.64	1.21	1.61	
		Reference	(1.42, 1.88)***	(1.05, 1.40)**	(1.42, 1.84)***	
	Middle	1.00	1.50	1.11	1.83	
		Reference	(1.30, 1.74)***	(0.97, 1.28)	(1.61, 2.09)***	
	High	1.00	1.51	1.14	1.89	
		Reference	(1.25, 1.81)***	(1.00, 1.29)	(1.63, 2.20)***	
Urbanicity						0.023
	Rural	1.00	1.59	1.19	1.60	
		Reference	(1.38, 1.83)***	(1.04, 1.35)**	(1.41, 1.81)***	
	Urban	1.00	1.55	1.11	1.86	
		Reference	(1.38, 1.73)***	(1.00, 1.23)*	(1.68, 2.07)***	
**Any cognitive disabilities**						
Sex						0.248
	Male	1.00	1.76	1.12	2.04	
		Reference	(1.54, 2.01)***	(0.99, 1.27)	(1.81, 2.30)***	
	Female	1.00	1.80	1.26	1.94	
		Reference	(1.55, 2.10)***	(1.09, 1.46)**	(1.69, 2.24)***	
Wealth						0.011
	Low	1.00	1.75	1.20	1.87	
		Reference	(1.48, 2.05)***	(1.01, 1.42)*	(1.60, 2.17)***	
	Middle	1.00	1.88	1.21	2.26	
		Reference	(1.57, 2.24)***	(1.02, 1.44)*	(1.93, 2.65)***	
	High	1.00	1.71	1.13	2.03	
		Reference	(1.41, 2.08)***	(0.97, 1.31)	(1.72, 2.41)***	
Urbanicity						0.021
	Rural	1.00	1.55	1.12	1.74	

© 2025 Wang Y et al. *JAMA Network Open*.

		Reference	(1.32, 1.82)***	(0.96, 1.30)	(1.52, 2.00)***	
	Urban	1.00	1.93	1.20	2.27	
		Reference	(1.70, 2.19)***	(1.06, 1.37)**	(2.01, 2.56)***	
**Any functional disabilities**[d]						
Sex						0.188
	Male	1.00	1.43	1.01	1.61	
		Reference	(1.34, 1.53)***	(1.03, 1.16)**	(1.52, 1.71)***	
	Female	1.00	1.47	1.21	1.58	
		Reference	(1.35, 1.60)***	(1.12, 1.31)***	(1.46, 1.71)***	
Wealth						0.175
	Low	1.00	1.47	1.21	1.61	
		Reference	(1.34, 1.61)***	(1.10, 1.32)***	(1.48, 1.75)***	
	Middle	1.00	1.43	1.10	1.67	
		Reference	(1.30, 1.56)***	(1.01, 1.20)*	(1.54, 1.81)***	
	High	1.00	1.46	1.10	1.59	
		Reference	(1.33, 1.61)***	(1.02, 1.18)**	(1.46, 1.72)***	
Urbanicity						0.065
	Rural	1.00	1.41	1.10	1.50	
		Reference	(1.29, 1.54)***	(1.02, 1.20)*	(1.39, 1.61)***	
	Urban	1.00	1.45	1.12	1.68	
		Reference	(1.36, 1.55)***	(1.06, 1.19)***	(1.59, 1.78)***	

Abbreviation: RR: relative risk

[a] Analyses weighted using 2018 ENSANUT survey-provided sampling weights.

[b] Data analyzed using modified Poisson regression analysis

[c] Adjusted for participant age, sex, education, marital status, ethnicity, urbanicity, region of residence, household asset score

[d] Any self-reported sensory, physical, and/or cognitive disabilities

*p<0.05, **p<0.01, ***p<0.001

© 2025 Wang Y et al. *JAMA Network Open*.
