# Supplementary material for: Food and Water Insecurity and Functional Disability in Adults
Source: JAMA Netw Open. 2025 Mar 20;8(3):e251271. doi: 10.1001/jamanetworkopen.2025.1271 (PMC11926644; doi:10.1001/jamanetworkopen.2025.1271)
Supplement: Supplement 2. — Data Sharing Statement [file jamanetwopen-e251271-s002.pdf]

Data Sharing Statement

Wang. Food and Water Insecurity and Functional Disability in Adults. *JAMA Netw Open*. Published online March 20, 2025. doi:10.1001/jamanetworkopen.2025.1271

Data
Data available: Yes
Data types: Other (please specify)
Additional Information: We used a public dataset for this study
How to access data: www.inegi.org.mx/programas/ensanut/2018/" >https://en.www.inegi.org.mx/programas/ensanut/2018/**When available:** With publication

Supporting Documents
Document types: None

Additional Information
Who can access the data: Anyone requesting the data
Types of analyses: For any purpose
Mechanisms of data availability: without investigator support
Any additional restrictions: None
